# Therapeutic Effects of Berberine on Liver Fibrosis are associated With Lipid Metabolism and Intestinal Flora

**DOI:** 10.3389/fphar.2022.814871

**Published:** 2022-03-02

**Authors:** Xianzhi Liu, Lifu Wang, Siwei Tan, Zebin Chen, Bin Wu, Xiaoying Wu

**Affiliations:** ^1^ Department of Gastroenterology, The Third Affiliated Hospital of Sun Yat-sen University, Guangzhou, China; ^2^ Guangdong Provincial Key Laboratory of Liver Disease Research, Guangzhou, China; ^3^ Department of Laboratory Medicine, The Third Affiliated Hospital of Sun Yat-sen University, Guangdong, China; ^4^ KingMed School of Laboratory Medicine, Guangzhou Medical University, Guangzhou, China; ^5^ Department of Hepatic Surgery, The First Affiliated Hospital of Sun Yat-sen University, Guangzhou, China

**Keywords:** cirrhosis, liver fibrosis, lipid metabolism, intestinal flora, berberine

## Abstract

Liver cirrhosis is a form of liver fibrosis resulting from chronic hepatitis caused by various liver diseases, such as viral hepatitis, alcoholic liver damage, nonalcoholic *steatohepatitis*, autoimmune liver disease, and by parasitic diseases such as *schistosomiasis*. Liver fibrosis is the common pathological base and precursors of cirrhosis. Inflammation and disorders of lipid metabolism are key drivers in liver fibrosis. Studies have determined that parts of the arachidonic acid pathway, such as its metabolic enzymes and biologically active products, are hallmarks of inflammation, and that aberrant peroxisome proliferator-activated receptor gamma (PPARγ)-mediated regulation causes disorders of lipid metabolism. However, despite the ongoing research focus on delineating the mechanisms of liver fibrosis that underpin various chronic liver diseases, effective clinical treatments have yet to be developed. Berberine (BBR) is an isoquinoline alkaloid with multiple biological activities, such as anti-inflammatory, anti-bacterial, anti-cancer, and anti-hyperlipidemic activities. Many studies have also found that BBR acts via multiple pathways to alleviate liver fibrosis. Furthermore, the absorption of BBR is increased by nitroreductase-containing intestinal flora, and is strengthened via crosstalk with bile acid metabolism. This improves the oral bioavailability of BBR, thereby enhancing its clinical utility. The production of butyrate by intestinal anaerobic bacteria is dramatically increased by BBR, thereby amplifying butyrate-mediated alleviation of liver fibrosis. In this review, we discuss the effects of BBR on liver fibrosis and lipid metabolism, particularly the metabolism of arachidonic acid, and highlight the potential mechanisms by which BBR relieves liver fibrosis through lipid metabolism related and intestinal flora related pathways. We hope that this review will provide insights on the BBR-based treatment of liver cirrhosis and related research in this area, and we encourage further studies that increase the ability of BBR to enhance liver health.

## 1 Introduction

Liver cirrhosis is a major global disease burden and leads to increased morbidity.(de Marco et al., 1999). The major causes of liver cirrhosis are viral hepatitis, alcoholic liver diseases and nonalcoholic fatty liver diseases, and some parasitic diseases. (Pinzani et al., 2011; Tsochatzis et al., 2014; Itaba et al., 2019). Liver fibrosis is the pathological hallmark and precursor of cirrhosis, and it is dependent on the activation of hepatic stellate cells (HSCs). (Tsuchida and Friedman 2017; Kisseleva and Brenner 2021). Recently, it has been determined that the balance between liver tissue regeneration and fibrosis, and their relationship with disorders of the liver microenvironment, play an important role in the pathology of liver fibrosis. (Poisson et al., 2017; Faillaci et al., 2018). Aberrant inflammatory processes in the liver and primary metabolic pathways in hepatocytes, together with intestinal flora, shape the liver microenvironment, with lipid metabolism playing a crucial role in this regard. Liver cirrhosis is thus a complicated, multi-phase, multi-pathway disease, whose pathogenesis remains to be fully characterized.

## 2 Mechanisms of Hepatic Stellate Cells Activation and Liver Fibrosis

Liver fibrosis is a wound healing process that is triggered by chronic liver damage. A central event of fibrogenesis is the *trans*-differentiation of quiescent HSCs to a myofibroblastic phenotype. (Friedman 2008; Wilson et al., 2015). Vitamin A-rich, lipid-storing HSCs show increased proliferative activity and fibrotic potential when activated by various types of liver stimuli. (Iredale 2007; Ramachandran et al., 2012). These activated HSCs can also release inflammatory signals to maintain myofibroblast activity and recruit adjacent normal cells for further activation and extracellular matrix (ECM) deposition, resulting in liver metabolism dysfunction and intrahepatic reconstruction. (Tsuchida and Friedman 2017; Henderson et al., 2020; Ginès et al., 2021; Kisseleva and Brenner 2021). HSC activation is controlled by multiple mechanisms, such as Hedgehog signalling, nuclear factor kappa B (NF-κB) signalling and mitogen-activated protein kinase (MAPK) activity. (Hellerbrand et al., 1998; Choi et al., 2009; S et al., 2009; Kim et al., 2017). Abnormal endoplasmic reticulum (ER) stress, oxidative stress, autophagy and ferroptosis, accompanied by inflammasome-associated signals, are common features of fibrogenesis. (Koo et al., 2016; Kim et al., 2017; Yang et al., 2021; Yi et al., 2021; Zhang et al., 2021). However, the pathogenesis of HSC activation and liver fibrosis remains unclear.

A growing body of evidence has revealed that HSC activation and fibrogenesis are associated with the versatility of liver metabolism and the tightly controlled homeostasis of intestinal flora. The dysregulation of lipid metabolism often presents as an imbalance between lipid synthesis, uptake and oxidation, which in turn causes liver inflammation and fibrosis.(Moustafa et al., 2012; Ristić-Medić et al., 2013; Arain et al., 2017). Acetyl-CoA carboxylase (ACC) inhibition has been shown to perturb fatty acid β-oxidation and *de novo* lipogenesis to reduce the sources of energy for HSC activation. (Hernández-Gea and Friedman 2012; Trivedi et al., 2021). Interestingly, peroxisome proliferator-activated receptor-gamma (PPAR-γ) and sterol regulatory element binding protein-1c (SREBP-1c), which are markers of quiescent HSCs, have been shown to modulate the adipogenic programme and thereby regulate HSC activation. (Eberlé et al., 2004; Tsukamoto 2005; Tsukamoto et al., 2006; Shao et al., 2016). The activation of farnesoid X receptors (FXRs) can suppress HSC activation and liver fibrosis via the reduction of triglycerides. (Maloney et al., 2000; Fiorucci et al., 2004). Surprisingly, it has also been suggested that intestinal flora can serve as independent regulators of liver metabolism, thereby influencing the progression, prognosis and regression of liver fibrosis. (Wei et al., 2013). This insight has been conceptualised as the gut-liver axis and has been a focus of recent studies on fibrogenesis. (Albillos et al., 2020; Bajaj and Khoruts 2020). Chen et al. showed that compared to healthy patients, cirrhosis patients have higher proportions of pathogenic *Enterobacteriaceae* and *Streptococcaceae* and lower proportions of beneficial *Lachnospiraceae*. (Chen Y. et al., 2011). In addition, the experimental antibiotic-mediated reduction of intestinal flora has been shown to decrease the abundance of microorganisms in the liver microenvironment, thereby alleviating liver fibrosis. (Seki et al., 2007). Conversely, germ-free mice show more severe ECM deposition and liver fibrosis than normal mice. (Mazagova et al., 2015; Henderson et al., 2020). These results suggest that some intestinal flora are hepatoprotective and others are harmful, and that the dysbiosis of intestinal flora is a key driver of HSC activation and liver fibrosis. Thus, lipid metabolism and intestinal flora may warrant exploration as targets for drugs for the treatment of liver fibrosis.

Despite mounting histological evidence suggesting that liver fibrosis is reversible, (Ni et al., 2014; Mogler et al., 2015) no methods can completely halt the pathological process. Current drugs applied in liver fibrosis treatment are primarily based on anti-inflammation and oxidative stress with limited effects. (Czaja 2014). Therefore, there is an urgent need to develop and validate effective therapies that specifically target liver fibrosis. Data show that berberine (BBR) functions via multiple networks to protect liver, resisting fibrosis and improving metabolism. (Zhang BJ. et al., 2008; Sun et al., 2009; Kumar et al., 2015; Zhang et al., 2016). In this review, we examine the underlying lipid metabolism-related and intestinal flora-related mechanisms of the biological activity of BBR and its therapeutic potential against liver fibrosis.

### 3 Sources, Bioavailability and Pharmacokinetic Characteristics of Berberine and its Derivatives

BBR, or 2,3-methylenedioxy-9,10-dimethoxyprotoberberine chloride, is a quaternary ammonium salt with a molar mass of 336.36122 g/mol. (Caliceti et al., 2016). It is one of a group of benzylisoquinoline alkaloids found in plants of the genus *Berberis*, such as *B. vulgaris* (barberry), *Phellodendron amurense* (Amur cork tree), and *Coptis chinensis* (Chinese goldthread), and the latter two species are used in Chinese herbal medicines. (Yin et al., 2008). BBR has a plethora of biological activities, such as antibacterial, antiinflammatory, (Kumar et al., 2015) anticancer, (Pandey et al., 2008) antidiabetic, (Zhang Y. et al., 2008) and hypolipidemic (Kong et al., 2004a) activities. However, BBR self-aggregates, does not effectively permeate into tissues, is subject to efflux and hepatobiliary re-excretion, (Feng et al., 2015) and undergoes first-pass processing in the small intestine. Thus, BBR is poorly absorbed in the body, and has an absolute bioavailability of 0.36%. (Liu et al., 2010a). BBR is metabolized in the liver by oxidative demethylation, which is performed by the cytochrome P450 enzyme system (mainly by CYP2D6, CYP1A2 and CYP3A4), to yield four major phase I metabolites (demethyleneberberine, berberrubine, jatrorrhizine, and thalifendine) (Liu et al., 2016); these are subsequently glucuronidated via UDP-glucuronosyltransferase (UGT) to their corresponding phase II metabolites. (Guo et al., 2016; Liu et al., 2016). These BBR metabolites act on the same targets as BBR (e.g., AMPK and the low-density lipoprotein receptor (LDLR)) but with a lower potency. (Li et al., 2011a). Ultimately, BBR and its derivatives are excreted primarily by hepatobiliary and renal pathways. Thus, there is a need for effective strategies to improve the oral bioavailability of BBR to enable its effective use in clinical settings.

## 4 Anti-fibrosis Effects of Berberine in the Liver

**FIGURE 1:**
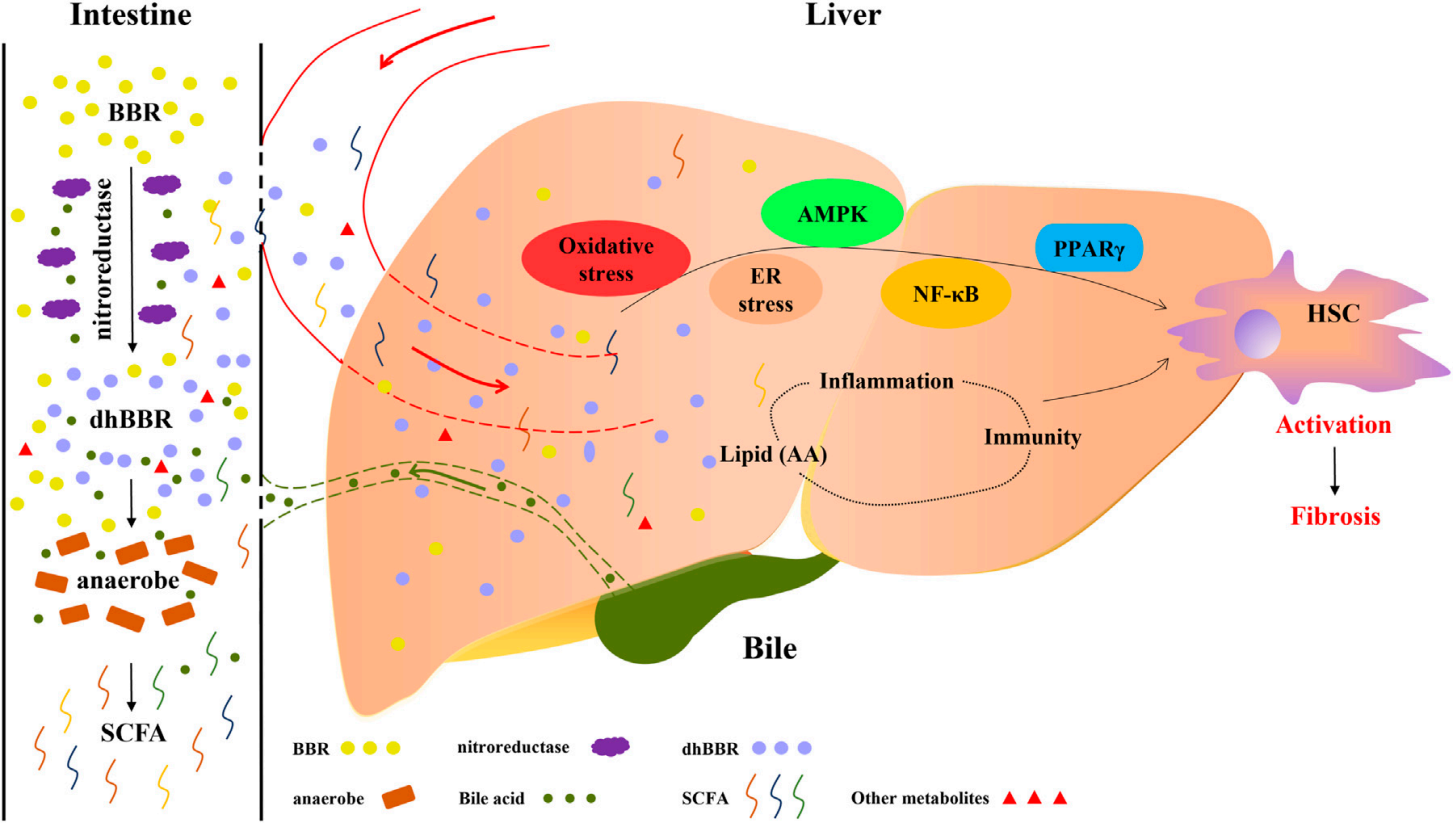
Therapeutic effects of berberine (BBR) on liver cirrhosis are associated with lipid metabolism and intestinal flora. BBR is converted to dihydroberberine (dhBBR) and other metabolites by the action of nitroreductase or specific intestinal microorganisms. dhBBR, other metabolites and unmetabolised pro-BBR in turn act on intestinal flora (such as anaerobes) to regulate the microorganism ecosystem and concentrations of intestinal metabolites, such as short-chain fatty acids. Unmetabolised pro-BBR, BBR derivatives and intestinal metabolites enter the liver through the portal vein, and thereafter relieve liver fibrosis by modulating lipid metabolism and regulating hepatic signalling. The potential mechanisms by which BBR reduces fibrosis include the regulation of oxidative stress, ER stress, AMPK, NF-κB and PPARγ signalling and the modulation of immune and inflammatory responses through the production of lipid mediators.

Several studies have demonstrated the efficacy of BBR against fibrotic diseases *in vivo*, including pulmonary fibrosis, (Chitra et al., 2015) myocardial fibrosis, (Zhang et al., 2014) renal fibrosis, (Wang et al., 2014) and adipose tissue fibrosis, (Xu X. et al., 2021) and multifaceted causal relationships illustrate the efficacy of BBR against liver fibrosis. (Wang et al., 2016; Bansod et al., 2021). As a multifunctional drug used in traditional Chinese medicine, berberine (BBR) can be used to treat various liver diseases. (Yang et al., 2021). The latest study from our team shows that BBR is a potential anti-liver fibrosis agent. In fibrotic mouse models, we found that BBR alleviates liver fibrosis by inducing ferrous-ion redox reactions to activate reactive oxygen species (ROS)-mediated ferroptosis in hepatic stellate cells, which suggests a possible strategy for the treatment of liver fibrosis. (Yi et al., 2021). Similar effects of BBR in carbon tetrachloride (CCl_4_)-induced liver fibrosis models were also demonstrated by other team recently. (Bansod et al., 2021). The activity of BBR against these multifactorial chronic diseases may be attributable to its multi-targeted mode of action. (Zhang et al., 2011). Inflammation and oxidative stress are key drivers of liver fibrosis, and it has been clearly demonstrated that BBR has anti-inflammatory and anti-oxidative activities. (Zhou et al., 2008; Jeong et al., 2009; Shang et al., 2010). It is therefore that the activity of BBR against liver fibrosis has been explored in many studies during recent years. (Zhang BJ. et al., 2008; Sun et al., 2009; Zhang et al., 2016). As shown in Figure 1, various mechanisms of action of BBR have been widely explored, such as its regulation of HSC activation, oxidative stress, inflammation, lipid metabolism, AMPK and ER stress, and NF-κB- and PPARγ-related signalling pathways.

### 4.1 Direct Effects of Berberine on Liver Fibrosis

The fundamental feature of liver fibrosis is the abnormal activation of HSCs, and BBR has been shown to be a potential treatment for thioacetamide (TAA)-, CCl_4_-, ethanol- and high cholesterol-induced liver fibrosis models; in these contexts, it likely acts by suppressing HSC activation and downregulating alpha-smooth muscle actin (α-SMA) and transforming growth factor-β1 (TGF-β1) levels. (Sun et al., 2009; Domitrovic et al., 2013; Eissa et al., 2018; Bansod et al., 2021). Previous studies have indicated that the direct beneficial effects of BBR involving modulation of the expression of multiple genes involved in HSC activation, cholangiocyte proliferation and liver fibrosis are linked to the downregulation of two important ribonucleotide molecules that promote liver fibrosis progression: microRNA34a and long noncoding RNA H19. (Wang et al., 2021). Another commonly reported mechanism is the induction of HSC cycle arrest in G1 phase, which inhibits HSC activation and prevents liver fibrosis. (Zhou et al., 2021). In addition, BBR has been revealed to have direct antifibrotic activity in bile duct ligation-induced liver fibrosis, due to its suppression of HSCs activation, and (partly) due to its inhibition of the AMPK signalling pathway. (Wang et al., 2016). However, other studies have found that BBR exerts hepatoprotective effects and prevents liver fibrosis by activating the AMPK signalling pathway. (Li et al., 2014; Wang et al., 2016; Bansod et al., 2021). BBR was also shown to activate the AMP-activated protein kinase (AMPK) pathway and inhibit macrophage polarisation and TGF-β1/Smad3 signalling, thereby alleviating tissue fibrosis. (Xu X. et al., 2021). ER stress may be another target of BBR treatment, and it has indeed been confirmed that a reduction in ER stress was the most logical explanation for the fact that BBR hinders the progression of hepatic steatosis to fibrosis. (Zhang et al., 2016). Moreover, BBR was shown to directly relieve liver injury-induced hepatic metabolic disorders by decreasing ER stress in hepatocytes (Yang et al., 2021), and the inhibition of Akt/FoxO1 signalling-mediated reduction of oxidative ER stress has been associated with BBR treatment of liver fibrosis. (Bansod et al., 2021). In other work, Zhang et al. reported that BBR prevents hepatic fibrosis by regulating the antioxidant system and lipid peroxidation in multiple hepatotoxic factor-induced fibrosis models, which was reflected by improved liver function, an increased antioxidant index and a decrease in fibrosis markers. (Zhang BJ. et al., 2008; Bansod et al., 2021). BBR-mediated normalization of liver function, suppression of inflammation, amelioration of ECM deposition and prevention of fibrosis correlate with NF-κB- and PPARγ-regulation. (Cao H. et al., 2018).

Many anticancer agents, such as methotrexate, (Sadeghian et al., 2018) doxorubicin (Zhao et al., 2012) and cyclophosphamide, (Germoush and Mahmoud 2014) are hepatotoxic (and thus cause hepatitis, steatohepatitis, liver cell necrosis, liver fibrosis or cirrhosis), and it is imperative to identify ways to limit this hepatotoxicity. It is therefore encouraging that anticancer drug-induced liver histopathological changes, including fibrosis, are significantly decreased by BBR treatment in animal studies. (Zhao et al., 2012; Germoush and Mahmoud, 2014).

Orally administered BBR displayed therapeutic effects in cirrhotic patients in a 1982 Japanese clinical study, with these effects being due to BBR inhibiting intestinal bacterial tyrosine decarboxylase. (Watanabe et al., 1982). Moreover, some randomized, placebo-controlled trials have found that BBR has positive effects in hyperlipidemic patients with virus hepatitis-related cirrhosis. (Riccioni et al., 2018). However, there are few clinical reports proving that BBR can alleviate cirrhosis, and properly designed clinical trials must be performed to determine this. To this end, our group is currently performing a randomized controlled trial to assess whether BBR can trigger the regression of hepatitis B-related fibrosis (ChiCTR1900023426), and our preliminary results are encouraging.

### 4.2 Effects of Berberine Metabolites on Liver Fibrosis

As the absolute bioavailability of BBR is extremely low (<1%) and more than half of the pro-BBR is not absorbed by the intestine, BBR is converted by intestinal flora into absorbable metabolites such as dihydroberberine (dhBBR), oxyberberine (OBB), canadine and other compounds. (ENRIZ et al., 2006; Liu et al., 2010a; Chen W. et al., 2011; Feng et al., 2015). Two of these metabolic products of BBR, dhBBR and OBB, exhibit superior anti-inflammatory and anti-oxidant functions compared to pro-BBR as they modulate intestinal flora and inhibit TLR4-MyD88-NF-κB and MAPK signalling, resulting in the reduction of levels of the pro-inflammatory cytokines tumour necrosis factor (TNF)-α, interleukin (IL)-17, interferon (IFN)-γ, IL-1β and IL-6 and immunoglobulin IgA. (Li et al., 2019; Tan et al., 2019; Li et al., 2020). Previous studies have revealed that dhBBR reduces inflammation via an NOD-like receptor pyrin domain-containing 3 (NLRP3) inflammasome-related mechanism, which likely reduces the release of caspase-1,apoptosis-associated speck-like protein (ASC) and IL-1β to inhibit pyroptotic cell death, which is a form of programmed cell death that occurs in liver fibrosis. (Xu et al., 2021a; de Carvalho Ribeiro and Szabo, 2021). dhBBR may even have better anti-sclerotic effects than BBR. (Chen et al., 2014).

It has been reported that OBB treatment enhances superoxide dismutase (SOD), catalase (CAT) and glutathione peroxidase (GSH-Px) activities and decreases reactive oxygen species (ROS), malondialdehyde (MDA) and myeloperoxidase (MPO) concentrations to reduce oxidative stress. Liver function recovery mediated by OBB might therefore hinder the progression of liver diseases and promote liver regeneration. (Dou et al., 2021). OBB has also been shown to ameliorate the pathological deterioration of adipocytes and hepatocytes via the AMPK pathway and stimulate energy expenditure to control lipid homeostasis at smaller dosages than BBR. Moreover, OBB was demonstrated to inhibit macrophage migration and trigger a phenotypic conversion of M1 macrophages to M2 macrophages to relieve the inflammatory burden of the liver. (Li et al., 2021). Surprisingly, the BBR derivatives dhBBR, canadine, stylopine and coptisine were reported to inhibit TGF-β1-induced collagen secretion in *vitro* fibrotic conditions and possess anti-inflammatory, anti-fibrotic, wound-healing promoting and cytoprotective activities. (Pietra et al., 2015).

In summary, the metabolites of BBR appear to have similar effects to those of BBR prodrug in terms of anti-inflammatory, anti-oxidant and anti-fibrosis activities. Moreover, the former appears safer and more efficacious than BBR itself. Thus, BBR and its derivatives must be examined in future research on liver fibrosis.

## 5 Berberine Alleviates Liver Fibrosis by Modifying Lipid Metabolism

Pharmacological and clinical evidence has clearly demonstrated the efficacy of BBR in the treatment of metabolic diseases, including non-alcoholic fatty liver disease and hyperlipidaemia. These effects are partly based on the regulation of various metabolic processes, such as the inhibition of lipogenesis and adipose tissue fibrosis and the mechanical reduction of hepatic steatosis. (Xu X. et al., 2021). The liver is a major site of lipid metabolism and there is a potential link between liver fibrosis and disorders of lipid metabolism. Moreover, the dysregulation of arachidonic acid metabolic pathways are partly responsible for disorders of the liver microenvironment, which lead to liver fibrosis or cirrhosis with various etiologies. (Hayashi et al., 2001; Song et al., 2008; Isomoto 2009; Ishihara et al., 2012; Ristić-Medić et al., 2013; Arain et al., 2017). Rather than only being a consequence of liver cirrhosis, dysfunctional lipid metabolism forms a vicious cycle with cirrhosis. (Moustafa et al., 2012; Gaggini et al., 2019). Changes in the lipid profiles of patients with chronic liver disease may indicate a progression towards fibrosis, and certain lipid profiles represent different stages of liver fibrosis. (Gaggini et al., 2019). Yang et al. recently reported that BBR improves lipid metabolic disorder in tunicamycin-induced liver injury. (Yang et al., 2021). BBR could also significantly reduce hepatic lipid accumulation by modulating fatty acid synthesis and metabolism to prevent the progression of non-alcoholic steatohepatitis and liver fibrosis. (Wang et al., 2021). Several latent mechanisms, such as oxidative stress (Zhang BJ. et al., 2008) and ER stress mechanisms, (Zhang et al., 2016; Yang et al., 2021) have been extensively explored for their roles in the ability of BBR and its metabolites to treat hepatic injury, progressive fibrosis and cirrhosis, but few details are known on the mechanism by which BBR regulates lipid metabolism in liver fibrosis patients.

The administration of BBR has been found to simultaneously mitigate the expression of genes related to lipogenesis, inflammation and fibrosis; this represents another possible mechanism underpinning the effect of BBR against liver fibrosis, and is likely related to hypolipidemic mechanisms.(Luo et al., 2019). Non-alcoholic fatty liver diseases (NAFLDs) are chronic progressive diseases, and approximately one third of NAFLD cases progress from hepatitis to non-alcoholic steatohepatitis (NASH) to liver fibrosis or cirrhosis. (Cicero et al., 2018). A meta-analysis of the efficacy of BBR in NAFLDs found that BBR delayed or repressed the fibrotic process in the development of NAFLDs by improving lipid parameters and alleviating hepatic steatosis. (Wei et al., 2016). Moreover, clinical findings have indicated that BBR increases liver function in hyperlipidemic patients with alcoholic liver cirrhosis or hepatitis C cirrhosis by creating a positive feedback loop with serum cholesterol, triglyceride and low-density lipoprotein-c (LDL-C), without causing any adverse effects. (Zhao et al., 2008a).

### 5.1 Regulation of Triacylglycerol Metabolism by Berberine

**FIGURE 2:**
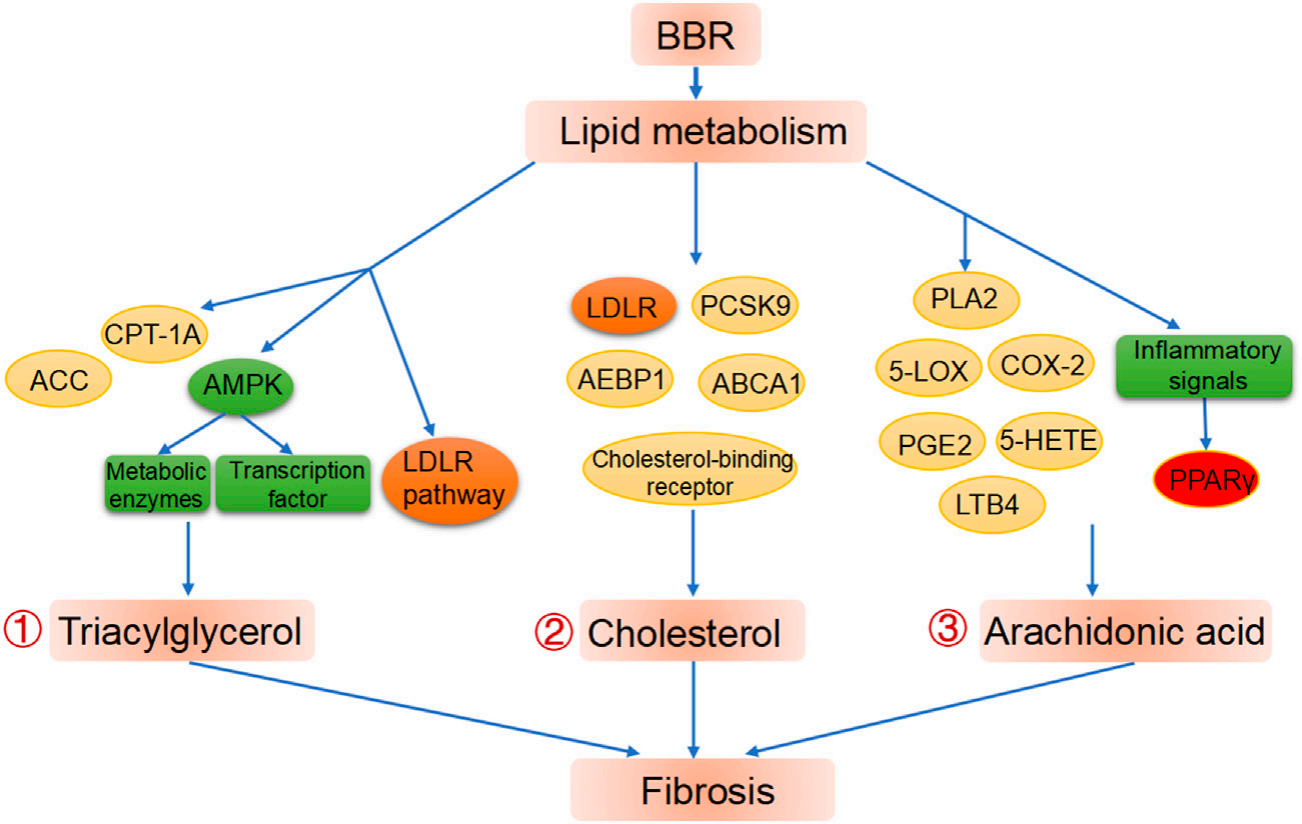
BBR improves liver fibrosis via lipid metabolism modification. ①Regulation of BBR on triacylglycerol metabolism. ②Regulation of BBR on cholesterol metabolism. ③ Regulation of BBR on cholesterol metabolism and PPARγ as a potential target in BBR treated fibrosis.

Elevated concentrations of triglycerides are a key contributor to lipid profile disorders and metabolic syndrome, and the ability of BBR to decrease hepatic and blood concentrations of triglycerides has been convincingly proven in both animal experiments and human studies.(Hu et al., 2012; Li et al., 2018). As such, BBR is used in many countries as a lipid-lowering drug for hyperlipidemia treatment. (Affuso et al., 2010). Animal experiments demonstrated that the pre-administration of BBR can reduce triglyceride accumulation in the liver caused by tunicamycin administration, thus treating liver injury. In particular, compared with the control group, the BBR group downregulated lipid metabolism-related gene expression of stearoyl-Coenzyme A desaturase 1 (SCD1). (Yang et al., 2021). The triglyceride-reducing efficacy of BBR is attributable to its decreasing triglyceride biosynthesis and increasing triglyceride oxidation. AMPK is essential for the control of lipid metabolism in terms of lipogenesis or lipid degradation, due to its affecting transcription factors and metabolic enzymes. (Fryer and Carling 2005). BBR also decreases the deposition of lipids in the liver by regulating AMPK, which balances fatty acid biosynthesis and oxidation. (Boudaba et al., 2018). Zhu et al. discovered that BBR can activate the AMPK-SREBP-1c pathway, which results in downregulation of the expression of hepatic stearoyl CoA desaturase 1 and other triglyceride (TG)-biosynthesis related genes, and in the attenuation of hepatic TG deposition, which alleviates NAFLD. (Zhu et al., 2019). Animal studies show that BBR can improve high fat diet-induced increases in serum triglyceride concentrations, thereby ameliorating hepatic steatosis and fibrosis via the SIRT3/AMPK/ACC pathway. (Yu-pei et al., 2019). Moreover, high-fat diet-induced hepatic steatosis is significantly inhibited by BBR treatment as reflected by the upregulated expression of proteins implicated in fatty acid oxidation, including ACC and carnitine palmitoyltransferase IA. (Zhang et al., 2019b). BBR can also regulate the LDLR pathway, by which BBR downregulates fatty-acid biosynthesis genes and upregulates fatty acid oxidation genes in adipocytes. (Lee et al., 2006; Lee et al., 2007). Xu et al. also found that BBR could improve systematic lipid homeostasis by promoting fatty acid β-oxidation; specifically, it causing deacetylation of long-chain acyl-CoA dehydrogenase via SIRT3 activation. (Xu et al., 2019). A meta-analysis of clinical trials was consistent with the evidence reviewed above, as it found that BBR lowered blood TG concentrations in a dose-dependent manner. (Dong et al., 2013). Therefore, the regulation of triacylglycerol metabolism may be a critical part of the mechanism of action of BBR (Figure 2).

### 5.2 Regulation of Cholesterol Metabolism by Berberine

The ability of BBR to decrease cholesterol concentrations was first described in human, animal and cell test in 2004, (Kong et al., 2004a) and BBR was subsequently found to have the same effect in diabetes mellitus (Zhang Y. et al., 2008) and cirrhosis (Zhao et al., 2008a) patients. It was also found that the phase I BBR metabolite berberrubine may decrease cholesterol concentrations by targeting LDLR expression. (Cao S. et al., 2018). The utility of BBR as a cholesterol-lowering drug has been consistently validated in clinical research, and it is widely used as a drug in Asian populations. Clinical trials and diverse disease models have been designed to confirm the beneficial effects of BBR on the regulation of cholesterol homeostasis. Abnormal cholesterol homeostasis was reversed to varying degrees after BBR intervention, and this was extensively probed in human studies and in hyperlipidemic and diabetic animal models. (Wang and Zidichouski 2018). Numerous randomized controlled trials have demonstrated that BBR supplementation improves blood profiles of total cholesterol, LDL-C, and high-density lipoprotein C (HDL-C), but some studies have not observed in HDL-C. (Kong et al., 2004a; Derosa et al., 2013; Wei et al., 2016). Moreover, the ability of statins to reduce cholesterol concentrations is significantly enhanced by BBR. (Moss and Ramji 2016).

The mechanisms by which BBR regulates cholesterol concentrations are related to anti-inflammatory processes or the post-transcriptional upregulation of LDLR expression.(Kong et al., 2004a; Pirillo and Catapano 2015). Proprotein convertase subtilisin/kexin type 9, which controls LDLR degradation, is inhibited by BBR, and is thus linked to the ability of BBR to decrease cholesterol concentrations. (Barbagallo et al., 2015). In addition, the adenosine triphosphate-binding cassette transporter A1 gene, which is involved in cholesterol efflux, is upregulated by BBR administration. (Lee et al., 2010). Similarly, BBR accelerates cholesterol excretion by inhibiting adipocyte enhancer-binding protein 1 (Huang et al., 2012) or augmenting cholesterol-binding receptor, which account for its hepatoprotective properties. (Zarei et al., 2015) (Figure 2).

### 5.3 Effects of Berberine on the Arachidonic Acid Pathway

Arachidonic acid is an essential unsaturated fatty acid that is stored under physiological conditions in cell membranes in the form of phospholipids. It is released from the phospholipid pool under various stimuli with the aid of phospholipase A2 (PLA2), and is subsequently converted into a wide variety of biologically active metabolites that induce the inflammatory cascade, such as 15-deoxy-Δ12,14-prostaglandin J2 (15 days-PGJ2), thromboxane B2 and prostaglandin E2 (PGE2). (Funk 2001). Cycloxygenase (COX), lipoxygenase (LOX) and cytochrome P450 (CYP450) are key enzymes in the metabolism of arachidonic acid. (Funk 2001; Calder 2009). Some metabolic enzymes involved in the arachidonic acid pathway can be inhibited by BBR, and this has been illustrated in various pathological processes. Specifically, BBR can affect the activity of metabolic enzymes such as PLA2(Huang et al., 2002; Li et al., 2015; Yarla et al., 2016a; Zhao et al., 2017), arachidonate 5-lipoxygenase (5-LOX) (Li et al., 2012) and COX-2 (Guo et al., 2008; Feng et al., 2012; Li et al., 2012; Wang and Zhang 2018), and in turn affects the production of downstream metabolites such as PGE2 and 5-hydroxyeicosatetraenoic acid to regulate disease course. (Huang et al., 2002; Zeng et al., 2011). A MetaboAnalyst system analysis showed that the arachidonic acid pathway affects biological activity of BBR in cancer interventions. (Li et al., 2017). For example, the anti-hepatocellular carcinoma effects of BBR involve inhibition of cytosolic PLA2 and COX-2. (Li et al., 2015). Extensive studies by various research groups have proven that BBR inhibits COX-2 expression and thereby decreases the production of PGE2. (Kuo et al., 2005; Singh et al., 2011). BBR is also a major element of a traditional Chinese medicine recipe, and in this form has been found to inhibit the expression of COX-2 and 5-LOX, and decrease the production of the inflammatory metabolites PGE2 and leukotriene B4, thereby ameliorating the effects of the inflammatory cascade. (Li et al., 2012).

Furthermore, studies of metabolic enzymes (particularly COX-2) have suggested that BBR benefits liver fibrosis in an arachidonic acid pathway-dependent manner. Domitrović et al. discovered that BBR provides protection against CCl_4_-induced liver injury in a concentration-dependent manner, which is partly attributable to a reduced COX-2 related-inflammatory response. (Domitrović et al., 2011). Similarly, the suppression of COX-2-driven inflammatory responses is also involved in the protective effects of BBR against drug-induced hepatotoxicity. (Germoush and Mahmoud 2014). Liver fibrosis is preceded by inflammation and can ultimately lead to liver cancer, and both of the latter have been widely reported to benefit from BBR treatment, due to its inhibition of the arachidonic acid pathway, (Domitrović et al., 2011; Germoush and Mahmoud 2014; Li et al., 2015; Yarla et al., 2016a; Zhang F. et al., 2019) which contains calcium-independent PLA2 and COX-2. These findings regarding the pathological development of chronic liver diseases and the biological function of BBR suggest that BBR and its derivatives could be used to treat liver fibrosis via arachidonic acid related mechanisms (Figure 2).

### 5.4 Peroxisome Proliferator-Activated Receptor Gamma as a Potential Target of Berberine

It is well accepted that PPARγ is a key molecule involved in the pathogenesis of liver fibrosis. (Zardi et al., 2013). PPARγ is encoded by the gene PPARG, and agonists of PPARG (e.g., IFC305 and pioglitazone) obstruct liver fibrosis by inhibiting HSC activation and regulating the expression of adipogenic- and fibrogenic-related genes. (Yuan et al., 2004; Perez-Carreon et al., 2010). PPARγ is also a crucial transcriptional regulator of genes involved in lipid metabolism, liver fibrosis, fat metabolism and adipocyte differentiation for adipose tissue development and functional maintenance. (Hazra et al., 2004; Ueki et al., 2004). Interestingly, BBR regulates lipid metabolism by precisely controlling the transcription and translation of nuclear reporters. (Zhou et al., 2008). PPARs have one of two different types of effects on fatty acid metabolic process, depending on their subtype. On the one hand, BBR inhibits TG production by acting with PPARα to enhance the expression of the gene coding for the fatty-acid oxidation enzyme carnitine palmitoyltransferase IA. (Zhou et al., 2008; Yu et al., 2016). On the other hand, PPARγ supports *de novo* fatty acid synthesis and fatty acid uptake. (Zhou et al., 2008). Zhou et al. showed that reduced PPARγ expression may be associated with the downregulation of adipogenic genes in the presence of BBR. (Zhou et al., 2008). High-throughput screening assays have also suggested that natural extract of BBR contains potential agonists of all PPAR subtypes (Xia et al., 2013; Tu et al., 2016; Yu et al., 2016) and that these can regulate the progression of liver diseases by acting as ligands. Interestingly, arachidonic acid metabolic products have also been reported to be PPARγ ligands and transcriptional activators (Xu et al., 1999; Choi and Bothwell 2012) that inhibit the activation of inflammatory signals, thereby modulating hepatic fibrosis via PPARγ regulation (Figure 2). The anti-fibrosis effect of PPARγ agonists (such as 15 days-PGJ2) has been observed in scarring models and has manifested as TGF-β-induced decreases in the extracellular matrix of HSCs. These findings imply that BBR acts on liver fibrosis via arachidonic acid pathway-mediated PPARγ activation. This is supported by research showing that arachidonic acid derived 15dPGJ2 attenuates fibrotic diseases by activating PPARγ, and that this effect is potentiated by co-administration of 15dPGJ2 and BBR. (Guan et al., 2018). Thus, it appears that PPARγ is a key target of BBR.

In conclusion, studies have confirmed the relationship between BBR, lipid metabolism pathways and subsequent signalling cascades, especially the arachidonic acid pathway. BBR may therefore relieve fibrosis by regulating PPARγ and restoring lipid homeostasis via modulation of arachidonic acid metabolism. More comprehensive studies on the effects of BBR on PPARγ, enzymes and downstream metabolites in the arachidonic acid pathway are needed to further elucidate appropriate clinical applications.

## 6 Contributions of Intestinal Flora to the Biological Function of Berberine

The regulation of intestinal flora by BBR application is a novel treatment strategy. BBR improves intestinal flora dysregulation and restores the gut barrier, effectively reducing plasma lipid concentrations and lipolysis. (Xu X. et al., 2021). BBR can also significantly reduce the levels of the opportunistic pathogens and increase the levels of probiotics. (Xu X. et al., 2021). With respect to the contributions of intestinal flora to the biological function of BBR in the treatment of liver diseases, Yang et al. showed that BBR alleviates tunicamycin-induced liver injury by regulating intestinal flora in mice, which it achieves by modulating the ratios of *Prevotellaceae* and *Erysipelotrichaceae* in the intestine. (Yang et al., 2021).

### 6.1 Intestinal Flora Improve the Efficiency of Berberine Absorption

**FIGURE 3:**
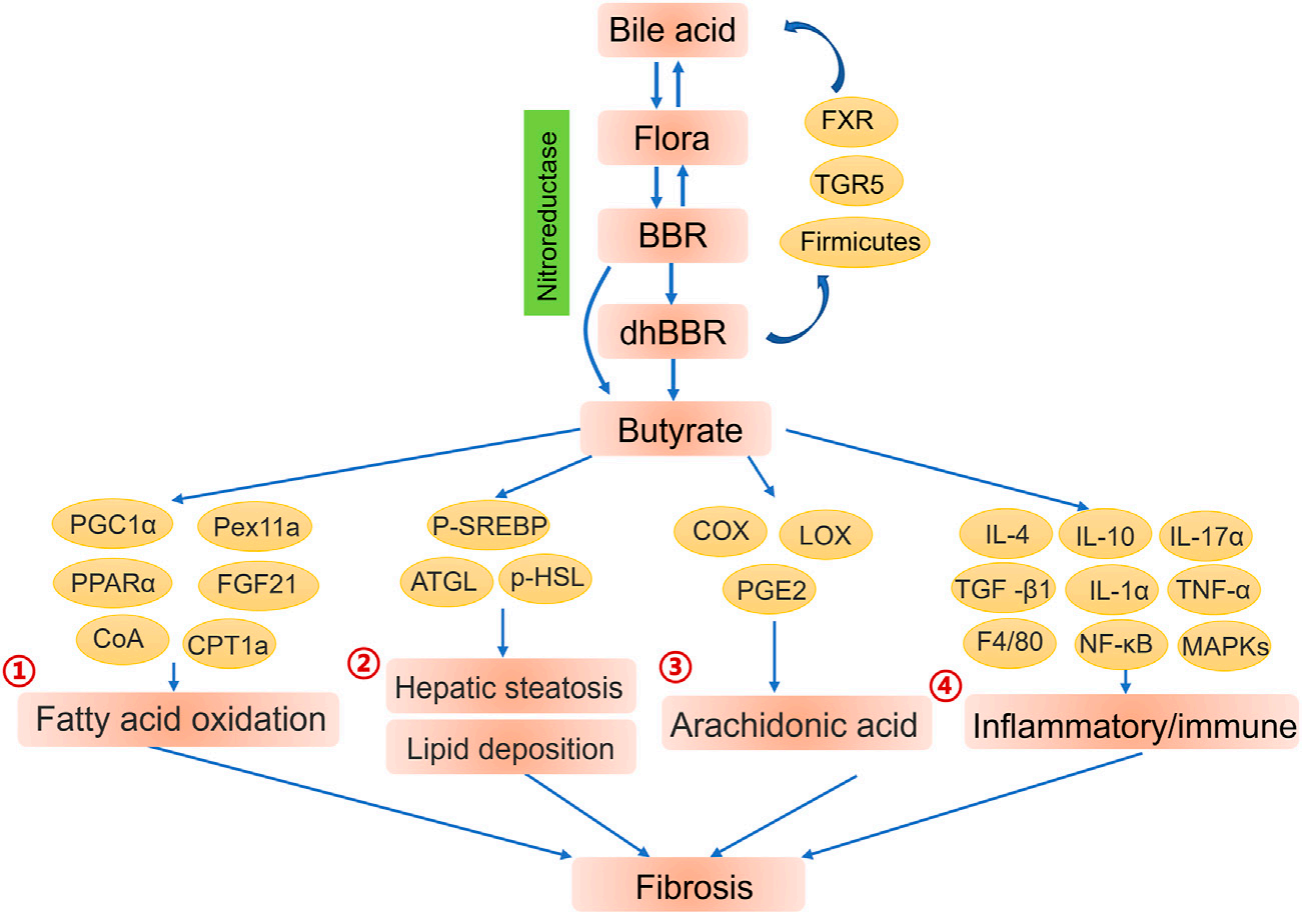
Contributions of intestinal flora to the biological function of berberine (BBR). Intestinal flora and bile acid improve the efficiency of absorption of BBR. BBR and its metabolites enhance the endocrine function of intestinal flora to further regulate the liver microenvironment and alleviate fibrosis. ①Butyrate enhanced fatty acid oxidation by activating PGC1α, Pex11a, PPARα and PPARα-mediated FGF21. (Weng et al., 2015; He and Moreau 2019). ②AMPK-dependent phosphorylation of SREBP (Li et al., 2011b) and enhancive expression of ATGL and phosphorylation of HSL (Jia et al., 2017) also offer promising pathway for butyrate to alleviate hepatic steatosis and lipid deposition through lipogenesis breakdown and lipolysis promotion.③ Butyrate treatment obviously inhibited arachidonic acid metabolism by altering the expression of metabolic enzymes (COX, LOX) together with synthesis of arachidonic acid metabolites (PGE2). (Ardaillou et al., 1985; Kamitani et al., 1998). ④Butyrate mediated inflammation remission and further liver fibrosis alleviation via promoting anti-inflammatory cytokines IL-4, IL-10 and inhibiting inflammatory genes TGF -β1, IL-1α, IL-17α, TNF-α, F4/80. (Ye et al., 2018).

Although the oral bioavailability of BBR is limited, intestinal flora promotes the absorption and enhance the efficacy of BBR. The BBR metabolites generated by intestinal flora are considered to be crucial to the biological activity of BBR; in particular, dihydroberberine (dhBBR), which has less biological activity than BBR but approximately five times the intestinal absorption rate of BBR. (Feng et al., 2015). Thus, the conversion of BBR to dhBBR, which is catalysed by nitroreductase, is the rate-limiting step that controls the amount of BBR entering the blood. Nitroreductase is present in many intestinal bacteria, such as *Staphylococcus aureus*, *Enterococcus faecium*, *Lactobacillus casei* and *L. acidophilus*. (Feng et al., 2015). The role of intestinal nitroreductase in potentiating BBR bioavailability is supported by the fact that BBR has greater efficacy in individuals with higher nitroreductase activity. (Wang et al., 2017b). Moreover, BBR increases the populations of probiotics containing nitroreductase, such as *Clostridia*. (Lemmon et al., 1997; Cui et al., 2018). After entering intestinal tissues, dhBBR is immediately reoxidised to BBR. (Feng et al., 2015). These findings indicate that intestinal flora derived nitroreductase may be a biomarker of the therapeutic efficacy of BBR (Figure 3).

### 6.2 Crosstalk Between Bile Acid and Intestinal Flora

BBR also restores bile acid homeostasis by targeting multiple pathways that markedly inhibit inflammation, thereby alleviating non-alcoholic steatohepatitis and liver fibrosis. (Wang et al., 2021). Bile acids serve as key regulators of lipid and glucose homeostasis, energy consumption and inflammation. (Yuan and Bambha 2015). Additionally, bile acids play critical roles in the homeostasis of intestinal flora, which may in turn regulate the size and composition of the bile acid pool that maintains normal bile acid excretion and hepatoenteral circulation. (Hofmann 1999; Ridlon et al., 2006; Rajilic-Stojanovic 2013). However, abnormal biliary secretion results in the destruction of microfloral structure, which adversely effects the abundance of bacteria responsible for bile acid catabolism, resulting in the improper excretion and reabsorption of conjugated bile acid. (Hedenborg et al., 1991). Nuclear receptor FXR and cell-surface receptor Takeda G protein-coupled receptor 5 (TGR5) can alter bile acid-mediated metabolism by binding to bile acids. (Pathak et al., 2018). Thus, BBR continues to be pursued as a potential agonist of FXR (Sun et al., 2017) and TGR5 (Yang et al., 2016) binding of bile acids, as this may offer ways to increase the abundance of bacteria that promote the decomposition of conjugated bile acids and regulate bile acid signalling. Furthermore, BBR significantly increases the abundance of intestinal Firmicutes, especially *Clostridium scindens*, which primarily maintain metabolism and the hepatoenteral circulation of bile acids. (Gu et al., 2015). Studies have revealed that the lipid-modification function of BBR is possibly achieved via the modulation of bile acid metabolism, (Sun et al., 2017; Meng et al., 2018) given that BBR regulates intestinal flora. (Gu et al., 2015). Thus, crosstalk between bile acid metabolism and intestinal flora might affect the absorption efficiency of BBR, which could be exploited in treatments for cirrhosis (Figure 3).

### 6.3 Berberine Enhances the Endocrine Function of Intestinal Flora to Further Regulate the Liver Microenvironment and Ameliorate Fibrosis

#### 6.3.1 Berberine Increases the Yield of Intestinal Flora Derived Butyrate

As mentioned, BBR is a promising candidate for the treatment of metabolic diseases by improving intestinal flora disorders.(Xu X. et al., 2021). It is currently thought that microflora function as a virtual “endocrine organ” (Clarke et al., 2014) that generates a wide variety of products to regulate host metabolism through homologous receptors. Short-chain fatty acids (SCFAs), particularly butyrate, acetate and propionate, which are the final products of the fermentation of indigestible carbohydrates by anaerobic microbes, exert profound effects on intestinal function and host energy metabolism. (Nicholson et al., 2012). The regulation of lipid profiles by BBR is realized not only via its direct effects on the blood concentrations of lipids, but also via its promoting the generation of SCFAs (mainly butyrate) to indirectly affect the blood concentrations of lipids. (Wang et al., 2017a). Zhang et al. proved this by demonstrating that concentrations of SCFAs in the intestine were increased by BBR treatment, which improved resistance to metabolic diseases. (Zhang et al., 2012). It has also been reported that BBR treatment leads to increases in the abundance of intestinal flora that secrete SCFAs and maintain host health, (Zhang et al., 2015) particularly *Clostridia*. (Gu et al., 2015; Byndloss et al., 2017; Cui et al., 2018).

#### 6.3.2 Effects of Butyrate on Lipid Metabolism

4-Phenylbutyric acid (PBA), a bioactive butyrate derivative with a long half-life, decreases ER stress and downregulates the transcription of numerous SREBP1-dependent lipogenic genes, which eventually leads to the inhibition of fatty acid biosynthesis. (Ren et al., 2013). However, butyrate also enhances fatty acid oxidation by activating peroxisome proliferator-activated receptor-γ coactivator 1-α, peroxisomal biogenesis factor 11 α, PPARα and PPARα-mediated fibroblast growth factor 21. (Weng et al., 2015; He and Moreau 2019). Moreover, butyrate-mediated ACC1 phosphorylation and inactivation not only inhibit fatty acid synthesis but also promote fatty acid oxidation by relieving malonyl CoA-induced carnitine palmitoyltransferase IA suppression. (McGarry et al., 1977; Hillgartner et al., 1995; Heimann et al., 2015). Additionally, AMPK-dependent phosphorylation of SREBP, (Li et al., 2011b) enhancement of the expression of adipose triglyceride lipase and phosphorylation of hormone-sensitive lipase (Jia et al., 2017) are pathways by which butyrate can alleviate hepatic steatosis and lipid deposition by inhibiting lipogenesis and promoting lipolysis. In particular, butyrate treatment inhibits arachidonic acid metabolism and thus suppresses inflammation, whereas reductions in butyrate concentrations aggravate NASH via an arachidonic acid-induced exaggerated inflammatory reaction. (Zhuang et al., 2017; Ye et al., 2018). Moreover, the administration of butyrate alters the expression of metabolic enzymes (e.g., COX and LOX) and thus affects the biosynthesis of arachidonic acid metabolites (e.g., PGE2). (Ardaillou et al., 1985; Kamitani et al., 1998).

Butyrate has also been reported to improve impaired liver function and alleviate the progression of fibrosis, which has a protective effect in NASH via arachidonic acid metabolism regulation. (Ye et al., 2018). In contrast, another study found that SCFAs adversely affect lipid metabolism: Yu et al. showed that SCFAs, including butyrate, exacerbate lipid accumulation in 3T3-L1 cells (a type of adipocyte) by promoting the expression of lipogenic genes and proteins. (Yu et al., 2018). Overall, butyrate appears to decrease inflammation and improve lipid metabolism in the liver (Figure 3), but further studies are needed to fully characterize its mode of action.

#### 6.3.3 Effects of Butyrate on Inflammatory/Immune Reactions

Research has shown that butyrate acts as a histone deacetylase inhibitor or acts on signalling receptors to suppress inflammation and thus postpone the development of liver diseases.(Le Poul et al., 2003; Donohoe et al., 2012; Gill et al., 2018). Butyrate decreases inflammation and alleviates further liver fibrosis by promoting production of the anti-inflammatory cytokines interleukin 4 (IL-4) and IL-10, and by inhibiting the expression of the genes coding for the inflammatory molecules transforming growth factor β 1, interleukin 1α (IL-1α), IL-17α, tumour necrosis factor α and F4/80. (Ye et al., 2018). Butyrate also suppresses the phosphorylation of MAPKs, the activation of NF-κB and the expression of downstream inflammatory signalling, thereby inhibiting inflammatory responses. (Ohira et al., 2013). Yukihiro et al. studied the important reciprocal interaction between immunity and inflammation, and revealed that microbiota-derived butyrate regulates transcription of the forkhead box protein P3 gene, which is positively correlated with concentrations of SCFAs and numbers of regulatory T cells. This resulted in the inhibition of inflammatory responses and ameliorated the development of colitis in T-cell-abnormal mice. (Furusawa et al., 2013). Overall, the above findings indicate that excessive inflammation and immune dysregulation are largely responsible for disorders in the liver microenvironment that lead to liver fibrosis. Furthermore, the positive effects of butyrate on inflammatory and immune responses provide a reliable theoretical basis for the effects of BBR in liver cirrhosis therapy (Figure 3).

#### 6.3.4 Effects of Butyrate on Liver Fibrosis

Researchers are increasingly exploring the ability of intestinal bacteria derived butyrate to alleviate liver fibrosis. For example, it has been found that the progression of fibrosis in methionine choline deficient diet induced NASH mice is substantially slowed by butyrate treatment, evidenced by a significant downregulation of the early fibrosis markers transforming growth factor-β1, smooth muscle α−actin and α-actin 2. (Ye et al., 2018). Butyrate’s effects on intestinal flora, lipid metabolism and inflammation have been proposed to underlie its effects in these mice. (Ye et al., 2018). Additionally, butyrate hinders the progression of NASH to fibrosis by regulating arachidonic acid metabolism. (Ye et al., 2018). These results indicated that butyrate may decrease liver fibrosis (Figure 3), but the mechanism of this remains to be fully delineated.

A balanced liver microenvironment is the basis for maintaining normal physiological functions, and an imbalanced liver microenvironment results in metabolic abnormalities, inflammatory activation and immune system perturbation. Butyrate produced by intestinal bacteria is absorbed through the intestinal mucosa, and then primarily distributed to the liver via portal veins, where it improves the liver microenvironment via mechanisms related to PPARγ activation. (Byndloss et al., 2017; Ye et al., 2018). Lipid metabolism and its interactions with inflammation and immunity may therefore account for the effects of butyrate treatment, and also create a link between BBR and cirrhosis. Thus suggests the possibility of a BBR–intestinal flora–butyrate–lipid metabolism–liver fibrosis interactive network.

## 7 Conclusion, Perspectives and Future Directions

BBR is a natural product with many useful biological effects and few adverse effects. Its effects on inflammatory and metabolic disturbances are particularly impressive. BBR has been confirmed to decrease liver fibrosis via multiple biochemical mechanisms, such as by regulating oxidative stress, ER stress, and the activity of AMPK, NF-κB and PPARγ (as shown in Figure 1). However, the complex mechanisms of action of BBR are not yet fully understood. Early studies on BBR highlighted its favorable effects on lipid profiles and interactions with inflammatory immune responses. We conclude from this review that BBR may exert its effects via the regulation of enzymes involved in arachidonic acid metabolism and downstream inflammatory pathways. Nevertheless, this has yet to be confirmed in cirrhosis models and further studies are warranted.

The poor oral bioavailability of BBR is a major hindrance to its clinical application. Fortunately, nitroreductase-containing intestinal flora or specific intestinal microorganisms can transform BBR into dhBBR, OBB, canadine and other derivatives, which are much more soluble and have better efficacy than BBR. These derivatives also have superior anti-inflammatory, anti-oxidant and anti-fibrosis functions, and bile acid metabolism has been shown to increase their formation via crosstalk with intestinal flora. BBR increases the production of butyrate by anaerobic bacteria, and the resulting higher concentrations of butyrate in circulation lead to improvements in host metabolism, decreases in inflammation, enhanced immunity and decreased liver fibrosis. The mechanism by which BBR promotes the metabolites of intestinal flora to further improve liver fibrosis by regulating the liver microenvironment remains largely elusive.

Beyond association studies, future research should develop a deeper understanding of the roles of the intestinal flora, arachidonic acid pathways and downstream targets (e.g., PPARγ) in liver fibrosis. Large-scale and multi-centre clinical trials are also required to verify the biological functions of BBR in cirrhosis. In addition, the safety, optimal dose and drug interactions of BBR must be taken into account. The bioavailability of BBR needs to be further improved by pharmaceutical techniques or medicinal chemistry approaches and by determining the precise mechanism of drug–host interactions.

This review summarizes current knowledge of the role of BBR in liver fibrosis in terms of its effects on lipid metabolism and intestinal flora. It is hoped that it will encourage future studies on BBR and lead to the development of novel strategies for the use of BBR in cirrhosis treatment, given the positive effects of BBR on liver fibrosis. Ultimately, this may yield personalized BBR-based approaches to treat liver fibrosis that are tailored to a patient’s unique intestinal microbiota profile.
